# First episode-psychosis: Short- and long-term outcomes and related features predicting the transition to schizophrenia

**DOI:** 10.1192/j.eurpsy.2022.2012

**Published:** 2022-09-01

**Authors:** M. Jabeur, L. Gassab, S. Boughamoura, A. Mhalla, F. Zaafrane, L. Gaha

**Affiliations:** Research laboratory LR 05 ES 10 “Vulnerability to Psychotic Disorders”, Faculty of medicine, University of Monastir, Psychiatry Department, University Hospital Of Monastir, Monastir, Tunisia

**Keywords:** First-episode psychosis, schizophrénia, risk factors, outcome

## Abstract

**Introduction:**

The occurrence of a first episode-psychosis in adolescents or young adults represents a difficult struggle with an uncertain and divergent outcome, since the clinician does not have at his disposal the clinical elements sufficient to predict these different disease trajectories.

**Objectives:**

Our aims are to describe the socio-demographic, clinical characteristics and the short and long-term outcomes of a first episode-psychosis and to identify the predictive factors of the transition to schizophrenia.

**Methods:**

We conducted a retrospective study about 117 patients hospitalized for a first episode-psychosis in the Psychiatric Department of Monastir (Tunisia). Sociodemographic and clinical features were collected using a pre-established form.

**Results:**

First-episode psychosis affected young male subjects with low educational level. Stressors were present in 54.7%. An 8-week prodromal phase preceded the onset of the disorder in 59%. The disorder course included diagnosis of: Brief psychotic disorder (32.5%), schizophrenia (31.6%) and bipolar disorder (18.8%). The short-term outcome was characterized by a complete remission rate of 58.1% at 3 months and 37.6% at 6 months. The long-term outcome was marked by a high rate of lost to follow-up: 70.8% after 5 years. The transition to schizophrenia was linked to the presence of delirium of influence and the absence of favorable course at 3 months.

**Conclusions:**

Our results led to the identification of the profile of patients with a first episode-psychosis and the factors correlated with a diagnosis of schizophrenia. Indeed, the determination of risk factors would make it possible to adapt earlier the care.

**Disclosure:**

No significant relationships.

